# Exploration of motivation to be physically active among overweight adolescents in Switzerland

**DOI:** 10.1177/13591045251315507

**Published:** 2025-01-15

**Authors:** Shahadije Sejdija, Albane BR Maggio

**Affiliations:** 1 27212 University of Geneva, Switzerland; 2 Health and Movement Consultation, Division of Pediatric Specialties, Department of Pediatrics, Gynecology and Obstetrics, 30512 Geneva University Hospitals and University of Geneva, Switzerland

**Keywords:** Motivation, adolescent, physical activity, prevention, obesity

## Abstract

Motivation plays an important role in the field of medicine, as it significantly influences behavior change, such as becoming more physically active. This study aimed to investigate the role of motivation in engagement in a physical activity and its impact on quality of life for adolescents suffering from obesity. We assessed the time dedicated to physical activities, the type of motivation, and the relationship between those factors and their quality of life. Seventy-two adolescents aged 12–18 years (51% girls) participated in the study. Fifteen percent of the sample (11/72) were overweight, 56% (*n* = 40) were affected by obesity and 29% (*n* = 21) by severe obesity. We found that adolescents were mostly driven to engage in physical activity by self-determined motivation, but those affected by severe obesity were more motivated by external factors. Moreover, there was a disparity in physical activity engagement between genders, with boys being more active than girls. Finally, our data demonstrated that physical exercise contributed to improving quality of life of our population. This study demonstrates that self-determined motivation plays a significant role in promoting physical activity among adolescents with overweight or obesity, with notable differences between genders. Encouraging intrinsic motivation can improve physical functioning and quality of life.

## Plain language summary

Motivation is a key factor in encouraging healthy behaviors, like being physically active. This study looked at how motivation influences physical activity and quality of life in adolescents with overweight or obesity. Researchers wanted to understand how much time these adolescents spend being active, what motivates them, and how this affects their well-being. The study included 72 adolescents with overweight or obesity aged 12 to 18. The study found that most adolescents were motivated to exercise by internal reasons (like personal goals or enjoyment). However, those with severe obesity were more influenced by external factors (like pressure from others). Boys were generally more physically active than girls. The study highlights the importance of encouraging self-motivation in adolescents with obesity. Helping them find personal reasons to stay activeâ€”rather than relying on external pressureâ€”can lead to better physical health and an improved quality of life. Programs that support self-motivation, especially for girls and those with severe obesity, may be particularly beneficial.

## Introduction

Obesity has become a significant global public health concern, with a steady increase in prevalence over the past 3 decades, including among children (Organization, 2021). In Switzerland, although the number of adults and children affected by obesity has remained stable according to the Federal Office of Public Health, a notable 17% of children are classified as overweight or obese (Stamm, Lamprecht, & Walter, 2021). Overweight and obesity have a significant impact on children’s mental health, often leading to mental health concerns, low self-esteem, social marginalization, and diminished quality of life (Förster et al., 2023). The associated social stigma and low self-esteem can contribute to the development of psychiatric disorders, such as anxiety, depression, and eating disorders in obese children (Nieman & LeBlanc, 2012; Smith, Fu, & Kobayashi, 2020). Evaluating the quality of life in this population is essential, as it provides a comprehensive understanding of how obesity influences a young person’s overall experience and may help detect adolescents at risk for mental health disorders. To prevent these comorbidities, it is crucial to identify and address the risk factors associated with obesity, particularly modifiable factors. Encouraging physical activity, reducing sedentary behavior and screen time, and promoting a balanced diet are essential strategies for preventing obesity-related comorbidities. By focusing on these factors and implementing motivational interviewing, we can work towards reducing the prevalence and impact of overweight.

Motivation plays an important role in medicine, significantly influencing behavior change. Intrinsic and extrinsic motivations have been described by Richard M. Ryan and Edward L. Deci (Deci et al., 1999; Ryan & Deci, 2000). Intrinsic motivation refers to the pleasure and interest derived from an action, independent of external reward. Understanding intrinsic motivation is crucial for fostering sustained engagement in physical activity and promoting overall well-being. In contrast, extrinsic motivation is driven by external factors that influence an individual’s behavior and can be imposed by others or the environment. These external factors include punishments, rewards, threats or social pressures, which can undermine autonomy and reduce intrinsic motivation (Deci et al., 1999). Extrinsic motivation can be divided into self-determined (e.g., integrated and identified motivation) and non-self-determined (e.g., external, and introjected regulation) types. Self-determined motivation includes actions aligned with personal values or recognized importance, while non-self-determined motivation is driven by external demand or guilt. In adolescents, motivation differs significantly from that of adults due to the distinct developmental stages and life priorities each group experiences. Adolescents are in a phase of identity formation, where social acceptance, peer influence, and self-esteem play crucial roles in shaping their motivation. They tend to be more externally motivated, often driven by the desire for social approval, peer recognition, or fear of judgment, as suggested by a recent study (Lou et al., 2023). They may shift towards more self-determined motivation as they gain autonomy and competence (Hu et al., 2021), but their ability to regulate long-term goals is still developing compared to adults, who are generally more adept at pursuing intrinsic goals and maintaining behaviors driven by personal values rather than immediate external rewards.

We hypothesize that adolescents will be mainly influenced by non-self-determined motivation. We speculate that their willingness to engage in physical activity is driven by peer comments, parental pressure, or medical advice, factors that may generate discomfort and body shame, potentially hindering their participation in sport.

Our study aimed to assess the self-reported interest of overweight and obese youth in engaging in physical activity, considering both intrinsic and extrinsic factors. Additionally, we explored the relationship between motivation subtypes, the amount of physical activity, and the impact on participants’ overall quality of life.

## Materials and methods

### Study design and subjects

This prospective observational study was carried out at our specialized obesity consultation center between October 2022 and March 2023. French-speaking adolescents aged 12–18 years, with a body mass index (BMI) above the 90^th^ percentile (overweight) according to the WHO growth charts, were recruited. During the visit, we collected relevant demographic and anthropometrics data, and adolescents completed three questionnaires under the supervision of a doctor or nurse.

The study was approved by the Cantonal Ethics Committee (CCER 2022-01419), and written informed consent was obtained from parent or adolescent when older than 14 years old.

### Measurements

#### Demographics and anthropometrics

We collected the participants’ age and gender, along with body weight (kg) in light clothing (underwear and a t-shirt) and height (cm) without shoes. Measurements were taken using a calibrated medical scale and stadiometer by a trained nurse. Body weight was measured once, while height was measured twice, with the higher value recorded. Body mass index (BMI) was calculated as weight/height squared (kg⋅m^−2^), and z-scores were derived using the World Health Organization references (WHO Multicentre Growth Reference Study Group, 2006). Overweight was defined as a BMI z-score > + 1SD, obesity as > + 2SD and severe obesity as > + 3SD.

#### Behavioral regulation in exercise questionnaire (BREQ-2)

The survey consists of 24 questions, divided into 6 subtypes of motivations: intrinsic, integrated, identified, introjected, external, and amotivation. Each subtype is assessed by four questions, with participants rating their agreement on a Likert scale from 1 (Not true at all) to 7 (Very true). These responses are used to calculate scores for each motivation subtype. Based on these scores, we were able to analyze the three types of motivation for each participant: self-determined motivation (including the intrinsic, integrated and identified subtypes), non-self-determined motivation (introjected and external subtypes) and amotivation. The BREQ-2 is a reliable and valid tool for measuring exercise motivation, with a Cronbach’s alpha coefficients ranging from 0.73 to 0.86, depending on the population studied (Markland & Tobin, 2004; Wilson et al., 2006). Factor analysis supported the distinctiveness of integrated regulation as a key factor in exercise motivation (Wilson et al., 2006). Furthermore, after the inclusion of the new amotivation subscale, the factor analysis results supported the expected five-factor structure of the questionnaire, confirming the factorial validity of the BREQ-2 (Markland & Tobin, 2004). However, neither study provided detailed test-retest reliability data. (French version of the BREQ-2 questionnaire in the supplementary files or online: https://onaps.fr/wp-content/uploads/2020/10/Calcul-Breq-2.pdf).

#### Physical activity questionnaire

We used the French adaptation of the Youth Risk Behaviour Surveillance System (YRBSS) questionnaire for children aged 11–14 years old, applying it to all participants regardless of age (The questionnaires and a description of the calculation method can be found in the supplementary files). This survey assessed the participants’ physical activity levels over the previous week, allowing us to calculate the minimum amount of physical activity performed. Additionally, it provided insights into the sedentary behavior of the adolescents. Although the YRBSS is considered reliable and valid in older populations (14–18 years old) (Brener et al., 2002), it has not been evaluated specifically in the 11 to 14-year-old age group. Test-retest reliability has been shown to range from moderate to substantial, with most kappa values falling between 0.41 and 0.81, depending on the specific question. Percent agreement is generally high, with most items showing agreements above 70%. Internal consistency reliability has not been evaluated.

We also collected data on the amount of time spent seated, whether at school or during screen use. Furthermore, we gathered information about their transportation habits, including time spent and the modes of transportation used.

#### Quality of life questionnaire PedsQL TM 4.0-SF-15 (13–18 years old version) (J. W. Varni, M. Seid, & P. S. Kurtin, 2001)

This survey consists of 15 questions divided into four domains of functioning: physical, emotional, social, and school (Varni et al., 2001a). Each domain includes a specific number of questions (5 for physical, 4 for emotional, 3 for social and 3 for school). Participants responded using a Likert scale, ranging from zero (Never) to 4 (Almost always). Following the provided instructions, we calculated functioning scores for each domain by summing up the responses. These domain scores can also be combined into a psychosocial functioning score, which includes the emotional, social, and academic domains. Additionally, a physical functioning score was calculated by summing up all the relevant domains related to physical well-being. Furthermore, a global quality of life score, ranging from zero to 100, was determined for each participant, with higher scores indicating better quality of life. The PedsQL 4.0 SF-15 has demonstrated high internal consistency reliability, with Cronbach’s alpha coefficients for the total score exceeding 0.80 (Varni et al., 2001b). Test-retest reliability has been demonstrated to range from 0.46 to 0.73 for child report in a population of children aged 8 to 12 attending school in Japan (Chen et al., 2007). Construct validity, evaluated by the known-groups method, has shown good differentiation between healthy children and acute or chronic health conditions, and factor analysis, performed with multitrait-multimethod (MTMM) analysis, is standing in the medium to large effect size range.

### Statistical analysis

Statistical analyses were performed using the SPSS software 25.0 (Chicago, IL). Descriptive analyses included frequency distributions for qualitative variables and mean and standard deviation (SD) for quantitative variables. Statistical differences between variables were analyzed using the independent Student’s t-test and the chi-square test (χ^2^) when appropriate. As some variables were not normally distributed, we assessed the relationship between them using non-parametric correlations (Spearman’s rho). Differences were considered significant if *p* < .05.

The sample size was calculated by estimating the confidence interval for a classical correlation coefficient using the Spearman method. Given the possibility that our results might follow a non-parametric distribution, we considered the following values: a correlation coefficient (rho) of 0.8, a confidence interval width (omega, ω) of 0.2, and a desired confidence level (alpha, α) of 0.05. By employing the critical value from the normal distribution, zα/2, which equals 1.960, we determined a 95% confidence interval of [0.676; 0.880], resulting in a width of 0.204. Based on this estimate, we determined that the minimum of participants required to obtain statistically significant results was 71.7724 (rounded up to 72). Therefore, we concluded that our study required at least 72 participants to draw reliable conclusions regarding the correlation between the various scores being examined.

## Results

### Patients characteristics

In this study we recruited 72 patients aged 12–18 years, with a mean age of 13.9 ± 1.5 years. Among these participants, 51% were girls. The mean body mass index was 31.2 ± 5.4 kg/m^2^, and the mean BMI z-score was 2.6 ± 0.6. Fifteen percent of the sample (11/72) were overweight, 56% (*n* = 40) were affected by obesity, and 29% (*n* = 21) by severe obesity.

When analyzing the differences between genders, we found no statistically significant difference in terms of age, but the BMI z-score was significantly higher in boys (independent *t* test BMI z-score in boys: 2.8 ± 0.6; in girls: 2.4 ± 0.6; *p* = .004). Additionally, our study found that boys were more frequently affected by severe obesity (16 boys compared to 5 girls; χ^2^ test, *p* = .008).

### Motivation questionnaire

The collected data indicate that most adolescents had a higher self-determined motivation score compared to the other two scores (Table 1). Boys showed a higher self-determined motivation score than girls (independent *t* test: 66.7 ± 12.9 vs. 59.0 ± 14; *p* = .026). Regarding the other two scores, no statistically significant difference was found between gender. Furthermore, we observed a positive correlation between BMI z-score and self-determined motivation score (Spearman’s rho: r = 0.238, *p* = .047); however, this result was only observed in boys (boys: r = 0.455, *p* = .006; girls: r = 0.088, *p* = .615). The age of the participants was not correlated with any of the motivation scores.

**Table 1.** Results of the Behavioural Regulation in Exercise Questionnaire (BREQ-2).

	Mean (M)	Range	SD
Individual motivation scores			
Intrinsic	21.59	9.00-28.00	5.37
Integrated	18.42	5.00-28.00	5.68
Identified	22.32	7.00-28.00	4.87
Introjected	14.49	5.00-26.00	5.61
External	10.00	4.00-22.00	5.09
Global scores			
Amotivation score	6.96	4.00-17.00	3.62
Self-determined motivation score	62.90	21.00-84.00	14.29
Non-self-determined motivation score	24.60	9.00-48.00	8.59

Self-determined motivation score includes intrinsic, integrated, and identified subtypes.

Non-self-determined motivation score includes introjected and external subtypes.

### Physical activity questionnaire

The results regarding physical activity for all subjects are presented in Table 2. We did not find any difference between boys and girls concerning the screen time during weekdays or weekends. However, we observed that boys spent more time engaging in physical activities compared to girls, with an average of 342.6 ± 156.3 minutes for boys versus 245.0 ± 157.6 minutes for girls (independent *t* test: *p* = .011).

**Table 2.** Results of Youth Risk Behaviour Surveillance System (YRBSS) Physical Activity Questionnaire.

	Mean	Range	SD
Minimal time of physical activity performed per week (minutes)	293.10	0.0-700.0	163.38
Time spent per day in front of screens on weekdays (hours)	4.22	1.00-15.05	2.92
Time spent per day in front of screens on weekends (hours)	6.75	2.0-20.00	4.06
Time spent per day walking or cycling (hours)	0.86	0.00-5.00	1.08

Furthermore, we observed that adolescents affected by obesity spent more time in active transportation (walking or cycling) compared to those who were overweight (independent *t* test: 0.94 ± 1.2 hours vs. 0.47 ± 0.4; *p* = .021). However, no correlation was found between BMI z-score and the amount of physical activity (Spearman’s rho: r = 0.159, *p* = .185). We also noted that the age of the participants was not correlated with any of the studied outcomes.

### Quality of life questionnaire

Concerning the results on quality of life, only 68 out of the 72 recruited adolescents were included in the total score calculation due to missing data from 4 participants. However, we decided not to exclude these individuals from the overall analysis, as the quality-of-life questionnaire was not the primary objective of our study.

The quality-of-life results are presented in Table 3. Girls had a lower overall quality of life score than boys (independent *t* test: 67.8 ± 13.9 vs. 75.2 ± 13.4; *p* = .029). This difference was primarily driven by lower scores in the domains of activities (74.6 ± 14.3 vs. 82.3 ± 14.9, *p* = .032) and emotions (56.6 ± 25.1 vs. 72.9 ± 16.6, *p* = .004) for girls compared to boys. Additionally, overweight youth had a better score in physical functioning than those with obesity (85.5 ± 6.9 vs. 77.3 ± 15.; *p* = .010).

**Table 3.** Results of Quality of Life Questionnaire PedsQL TM 4.0-SF-15.

	Mean	Range	SD
Activities score	78.5	35.0-100.0	15.0
Emotional functioning score	64.9	0.0-100.0	23.8
Social functioning score	80.0	8.5-100.0	24.5
School functioning score	58.3	0.00-100.0	22.2
Psychosocial functioning score	67.9	20.1-97.9	17.0
Physical functioning score	78.5	35.0-100.0	15.0
Overall quality of life score (*n* = 68)	71.6	40.0-96.7	14.0

### Correlation between questionnaires

The results of our study indicate significant associations between physical activity, active transportation, motivation, and quality of life in our young participants. Firstly, it was observed that the more time young people devoted to physical activity, the more time they spent in active transportation (walking or cycling) (Spearman: *p* = .039). These two factors were independently and positively correlated with physical functioning (*p* < .05). Moreover, all three factors (physical activity time, active transportation time, and better physical functioning) were positively associated with self-determined motivation (Spearman: *p* = .003, *p* = .030 and *p* = .04, respectively).

Regarding non-self-determined motivation, it was found to be associated with lower scores in the domains of quality of life. Specifically, extrinsic motivation was negatively correlated with psychosocial, physical functioning, and the overall quality of life score (Spearman’s rho: r = 0.377, *p* = .002; r = 0.274, *p* = .024; r = 0.417, *p* < .001; respectively). However, extrinsic motivation was not related to physical activity or screen time. Furthermore, amotivation was statistically associated with lower scores in physical functioning (r = 0.328, *p* = .006), and there was a trend towards an association between amotivation and lower overall quality of life score (r = 0.237, *p* = .054).

Finally, we observed a lower psychosocial functioning score among young people who spent more time in front of screens during the week (r = 0.265, *p* = .039) and among those with lower physical functioning scores (r = 0.432, *p* < .001).

## Discussion

This study contributes to the existing literature by exploring the role of self-determined and non-self-determined motivation in physical activity among adolescents with overweight or obesity. Contrary to expectations, the findings revealed that most participants demonstrated higher level of self-determined motivation rather than non-self-determined motivation. This differs from previous assumptions that adolescents with obesity would primarily be influenced by external factors such as peer pressure, parental expectation, or medical advice, which could discourage their engagement in physical activity. These results align with earlier research but extend the literature by specifically considering the impact of obesity on motivation. Indeed, a study conducted in Spain highlights that self-determined motivation is the main driver of physical activity in most children and adolescents (Romero-Parra et al., 2023). However, unlike our study, they didn’t analyze the impact of obesity on their results. Our analyses showed a positive correlation between a high BMI z-score and a non-self-determined motivation. These results support our initial hypothesis, suggesting that adolescents with obesity may be motivated to remain active primarily due to external pressures, such as parental expectations or negative influences from their peers, including feelings of body shame and guilt for not being active. Conversely, adolescents less affected by obesity appear to be more motivated by self-determined reasons. According to the meta-analysis by Deci, Koestner and Ryan (Deci et al., 1999), for an adolescent to engage in physical activity, they must feel competent. However, competence alone is not sufficient; it must be coupled with a sense of autonomy. If adolescents participate in an activity driven by extrinsic motivation, they are likely to abandon it over time. Therefore, it is essential for them to develop a genuine personal interest in the activity, allowing them to experience cognitive, social, and physical satisfaction, which will enhance their overall engagement.

Secondary findings highlighted notable gender differences. Boys exhibit higher level of self-determined motivation, physical activity, and quality of life compared to girls, consistent with prior studies. According to the authors of the study conducted in Spain, girls were more likely to perceive sports as competitive or masculine and feared embarrassment when engaging in physical activity, especially in mixed-gender settings. Furthermore, the SOPHYA study (Probst-Hensch & Bettina, 2014), showed that boys were nearly twice as likely to follow the WHO recommendations compared to girls. This raises concerns about girl’s participation and engagement in physical activities, as it could impact on their health and well-being. The SOPHYA study suggests that the under-representation of girls in sports clubs could potentially contribute to this disparity. Even though they were less active, girls did not appear to spend more time in sedentary behavior, as screen time did not differ between the two genders. Quality of life analyses showed significant differences between gender and weight status among adolescents in our study. Girls reported a lower quality of life than boys, particularly in aspects related to activities and emotions. These results suggest a correlation between staying active and improving quality of life, a finding also supported by the SOPHYA study, which demonstrated that physical activity positively impacts quality of life, lifestyle, and resilience to stress (Probst-Hensch & Bettina, 2014).

Furthermore, our study found that physical activity, active transportation, and self-determined motivation play a key role in physical functioning and quality of life among youth. This suggests that staying physically active and prioritizing active transportation may promote better health and physical capacity. Regarding sedentary time, our data suggest a negative impact on the psychosocial functioning of young individuals.

Finally, external motivation and amotivation were associated with a lower quality of life, underscoring the importance of self-determined motivation for achieving a better quality of life. Intrinsic motivation, driven by personal interest and enjoyment may be beneficial for overall well-being.

Regarding the selected sample, it can be said that it is representative of the population we aimed to study. Only four patients declined to participate in the study, but they received the same information as the participants. Through the recruitment process, we obtained an equal number of girls and boys of similar age, making the sample comparable and reducing potential biases. However, the only statistically significant difference observed is the disparity in body shape between boys and girls, with boys being more frequently affected by severe obesity.

The study has clinical implications, particularly in promoting physical activity among adolescents with overweight or obesity. Encouraging self-determined motivation, rather than focusing on external pressures, may improve long-term adherence to physical activity. Moreover, targeted interventions are needed to address the gender gap, by fostering a supportive environment for girls to develop competence and autonomy in physical activity. Active transportation should also be emphasized as a viable form of physical activity for adolescents with obesity.

This study has several limitations. The use of self-reported measures for physical activity and motivation may introduce bias and the sample size was relatively small. Future studies should incorporate objective measure of physical activity and include larger, more diverse sample to strengthen the validity of these findings.

Future research should focus on identifying more effective strategies to enhance self-determined motivation, especially for girls. Larger, longitudinal studies are required to confirm these findings and explore the long-term effects of motivation type on physical activity engagement, quality of life, and overall health outcomes in adolescents with varying weight statuses.

## Conclusion

This study demonstrates that self-determined motivation plays a significant role in promoting physical activity among adolescents with overweight or obesity, with notable differences between genders. Encouraging intrinsic motivation and active transportation can improve physical functioning and quality of life. Using motivational interview may be of valuable importance as youth suffering from obesity may be more open to external reasons to be more active as well as internal motivation. However, future research with larger, more diverse samples and objective measures is needed to confirm these findings and address the gender disparities in physical activity engagement.
